# Assessing Nanopore Targeted Sequencing for the Diagnosis of Pulmonary Infections: A Comparative Multidisease Approach

**DOI:** 10.1155/cjid/5479737

**Published:** 2026-02-03

**Authors:** Ying Zhao, Guannan Ma, Tianxiao Ma, Yongshuai Miao, Heya Na, Yuhui Liu, Xiaohua Li

**Affiliations:** ^1^ Affiliated Chifeng Clinical Medical College of Inner Mongolia Medical University, Chifeng, China; ^2^ Medical Research Center, Zhejiang Key Laboratory of Digital Technology in Medical Diagnostics, Hangzhou, China; ^3^ Department of Pulmonary and Critical Care Medicine, Chifeng Municipal Hospital, Chifeng, China, cfsyy.cn

**Keywords:** chronic obstructive pulmonary disease (COPD), conventional microbiological tests (CMTs), hypertension (HBP), nanopore targeted sequencing (NTS), pathogen detection, pulmonary infection

## Abstract

Pulmonary infections remain a significant global health burden, particularly in immunocompromised individuals and patients with chronic respiratory or systemic diseases. Conventional microbiological tests (CMTs), though widely used, often have limited sensitivity and delayed results, especially in polymicrobial or atypical infections. This study evaluated the diagnostic performance of nanopore targeted sequencing (NTS) in 284 patients with suspected lower respiratory tract infections at Chifeng Municipal Hospital, using CMTs and clinical diagnosis as references.

NTS demonstrated markedly higher sensitivity (91.85%) compared to CMTs (74.81%), with substantial improvements in detecting fungal (81.7%) and mixed infections (99.65%). A total of 259 pathogens were detected. Among them, bacteria were the most frequently identified pathogens (69.5%), followed by fungi (15.44%) and viruses (14.28%). Pathogen distribution varied by clinical subgroup, such as community‐acquired pneumonia and chronic obstructive pulmonary disease, reflecting infection heterogeneity. Patients with hypertension (HBP) showed a higher incidence of fungal and mixed infections than non‐HBP patients. NTS was particularly effective in detecting opportunistic pathogens in the HBP group, suggesting an association between cardiovascular comorbidity and altered pathogen susceptibility.

**Summary**

• NTS significantly outperformed CMTs in sensitivity and spectrum of pathogen detection, including organisms often missed by conventional methods. Its integration into diagnostic workflows may improve etiological precision and support personalized antimicrobial strategies, particularly in patients with comorbidities.

## 1. Introduction

Pulmonary infections are a major cause of morbidity and mortality worldwide, particularly among immunocompromised individuals and hospitalized patients [1]. These infections encompass a wide spectrum of diseases caused by bacteria, viruses, fungi, and mycobacteria, with pathogen distribution varying significantly depending on clinical settings and patient populations. For instance, community‐acquired pneumonia (CAP) is predominantly caused by *Streptococcus pneumoniae, Haemophilus influenzae*, *Mycoplasma pneumoniae*, and respiratory viruses such as influenza virus and SARS‐CoV‐2 [2]. In contrast, hospital‐acquired pneumonia (HAP) is caused by Gram‐negative bacteria (GNB) among bacterial pneumonia [3], including *Pseudomonas aeruginosa*, *Acinetobacter baumannii*, and methicillin‐resistant *Staphylococcus aureus* (MRSA), which pose significant challenges for empirical treatment due to high antibiotic resistance rates.

In immunocompromised patients, such as those with hematologic malignancies, solid organ transplantation, or HIV infection, opportunistic pulmonary infections are common [4, 5]. Additionally, patients with chronic lung diseases (such as cystic fibrosis [CF], chronic obstructive pulmonary disease [COPD], and bronchiectasis) frequently experience polymicrobial infections. *Haemophilus influenzae* (*H. influenzae*) and *Moraxella catarrhalis* (*M. catarrhalis*) are associated with acute exacerbation of chronic obstructive pulmonary disease (AECOPD) [6, 7].

Despite the clinical significance of pulmonary infections, traditional microbial culture (conventional microbiological tests [CMTs]), including culture‐based methods, serology, and PCR, has notable limitations. These approaches often have low sensitivity for slow‐growing or nonculturable pathogens, are time‐consuming, and may fail to detect polymicrobial infections, particularly in patients receiving prior antibiotic therapy [8]. Furthermore, traditional methods are inadequate for the rapid identification of emerging or rare pathogens, limiting their utility in guiding timely clinical decision making [9].

Nanopore targeting sequencing (NTS) has recently emerged as a promising diagnostic technology, offering real‐time sequencing, broad‐spectrum pathogen detection, and higher sensitivity compared to conventional methods [10]. NTS enables the simultaneous identification of bacteria, fungi, mycobacteria, and viruses, providing a more comprehensive approach to diagnosing pulmonary infections [11]. Several studies have demonstrated that metagenomic sequencing improves pathogen detection, particularly in polymicrobial infections and cases where standard diagnostics yield negative results [12–14]. However, the clinical performance of NTS in pulmonary infections, particularly its sensitivity, specificity, and agreement with CMTs and clinical diagnosis, remains inadequately explored.

This study evaluates the diagnostic value of NTS for pulmonary infections by comparing its pathogen detection with CMTs and clinical diagnosis. Analyzing 284 patients, we found bacteria were the most common pathogens, followed by fungi and viruses, with CAP (74.29%) being the predominant disease. NTS showed significantly higher sensitivity (81.7%) than CMTs (40%), especially in detecting complex infections. Pathogen distribution varied by infection type (e.g., CAP and COPD) and comorbidities (e.g., hypertension [HBP]), underscoring the need for precise diagnosis and personalized treatment. Our findings may contribute to optimizing pathogen detection strategies and guiding more effective antimicrobial therapy in clinical practice.

## 2. Materials and Methods

### 2.1. Patient Cohort

This observational study enrolled 284 patients with suspected lower respiratory tract infection (LRTI) who presented to Chifeng Municipal Hospital between June 2023 and September 2024. Demographic data, clinical characteristics, medical history, diagnostic and therapeutic interventions, radiologic and laboratory results, microbiological testing, pathology, and clinical outcomes were collected. All patients underwent bronchoscopy, and bronchoalveolar lavage fluid (BALF) was collected from the lesion site based on CT imaging. Then the BALF was aseptically aliquoted and subjected to CMTs and NTS, respectively.

### 2.2. CMTs

BALF samples were subjected to standard microbiological analyses, including Gram staining, culture for bacteria, mycobacteria, and fungi, and acid‐fast staining. Additional tests were conducted as clinically indicated, such as fungal immunofluorescence staining, galactomannan antigen testing for *Aspergillus*, and cryptococcal antigen detection.

## 3. NTS

Respiratory tract samples (placed in sterile sputum containers) are collected and immediately transported to a commercial laboratory (Hangzhou DIAN Medical Laboratory, Hangzhou, China) using dry ice for testing. For a detailed procedure of NTS testing, please refer to the attached document (Supplementary 1).

### 3.1. Bioinformatic Analysis

Real‐time basecalling and demultiplexing were performed using MinKNOW software. Reads with low quality were filtered out, and human host sequences were removed using Minimap2 against the human reference genome (hg38). Pathogen identification was performed using Centrifuge v1.0.3 with the NCBI nonredundant nucleotide (NT) database. When the data size of a sample reaches 50 Mb and the number of detected sequences for the internal control (*Lactococcus lactis*) exceeds 100, the sample is deemed to have passed the quality control, and further analysis is conducted to determine positive species.

## 4. Final Clinical Diagnosis

Each patient’s final diagnosis was established at discharge based on a comprehensive evaluation that included clinical presentation, radiologic findings, laboratory tests, pathology, and results from CMTs and NTS. Diagnoses were made jointly by two respiratory physicians. In case of disagreement, a panel of three experts reviewed the case to reach consensus. These diagnoses served as the reference standard for assessing the diagnostic performance of NTS and CMTs.

## 5. Results

### 5.1. Clinical Information

This study included 284 patients recruited from one hospital between June 2023 and September 2024, comprising 118 males (41.55%) and 166 females (58.45%). The clinical features are shown in Table 1. Ages ranged from 15 to 90 years, with a mean of 58.80 years. The most common symptoms were cough, sputum, and fever. Of the participants, 195 patients had one or more background diseases, with HBP being the most prevalent (*n* = 104, 36.62%). Posttreatment, 182 patients (64.08%) showed clinical improvement or complete cure, while four patients died. Among the 284 patients, 270 were classified as infection‐related, with pathogens being the primary cause of their lung infections. As shown in Table 1, CAP was the most common disease type (*n* = 211, 74.29%), followed by COPD (*n* = 37, 13.03%) and bronchitis (*n* = 24, 8.45%). Comorbidities such as HBP (36.62%), history of surgery and trauma (16.55%), and diabetes (8.45%) were also frequently observed.

**Table 1:** Demographic and clinical characteristics (*n* = 284).

Characteristics	Patients, *n* = 284
Gender	
Male	118 (41.55%)
Female	166 (58.45%)
Age (years%)	
Range	15–90
Average	58.80 ± 13.97
Infection types	
CAP	211 (74.29%)
Infection	43 (15.14%)
COPD	37 (13.03%)
Bronchitis	24 (8.45%)
Tuberculosis	23 (8.10%)
CB	20 (7.04%)
Bronchiectasis	18 (6.33%)
Cancer	18 (6.33%)
AECB	13 (4.58%)
Asthma	10 (3.52%)
ILD	15 (5.28%)
Major complications	
Hypertension	104 (36.62%)
History of surgery and trauma	47 (16.55%)
Diabetes	24 (8.45%)
Liver and renal diseases	21 (7.39%)
Malignancy or immunocompromised	18 (6.34%)
Coronary heart disease	14 (4.93%)
Cerebral infarction	10 (3.52%)
Cerebral hemorrhage	3 (1.06%)

### 5.2. Specimen Types and Pathogen Distribution in NTS

We analyzed the distribution of sample sources and pathogen types in this study, revealing key insights into the characteristics of pulmonary infections. The majority of the samples were obtained from BALF, accounting for 85.2% (242/284) of the total samples, followed by sputum with 11.62% (33/284) and hydrothorax contributing 3% (8/284) (Figure 1(a)). Only 1 sample was collected using a throat swab, representing less than 1% of the samples. The 259 pathogens identified were mainly bacteria, which accounted for 56.76% (147 cases) of the total infections, underscoring their dominant role in pulmonary infections. *Mycobacterium* species contributed to 12.74% (33 cases, including *Mycobacterium tuberculosis*, *Nontuberculous mycobacterium*, *Mycobacterium abscess*, and *Mycobacterium ornithine*), reflecting their importance in chronic and opportunistic infections. Fungal infections were identified in 15.44% (40 cases). Viral infections were also observed, with RNA viruses and DNA viruses comprising 7.72% (20 cases) and 6.56% (17 cases), respectively. *Mycoplasma*, *Chlamydia*, and spirochetes were less common, accounting for only 0.77% (2 cases) (Figure 1(b)).

Figure 1Distribution of sample sources (a) and pathogen types (b) of NTS.

**Figure:**
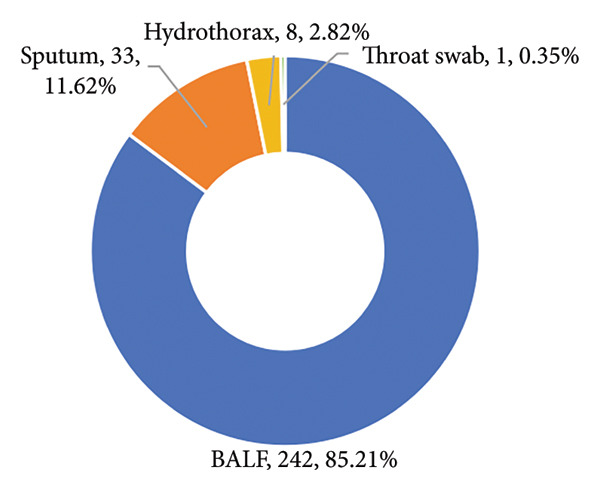
(a)

**Figure:**
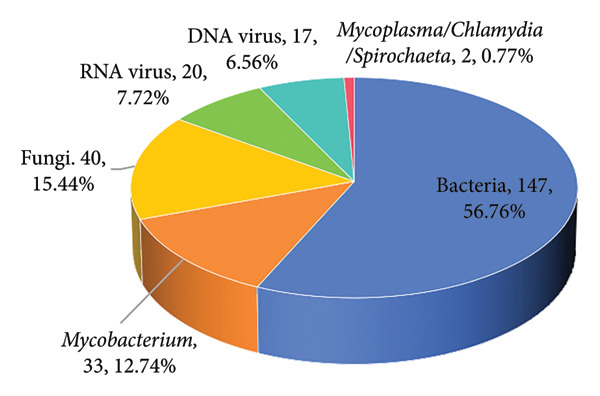
(b)

## 6. Comparison of Pathogen Detection Methods

A comparative analysis was performed to evaluate the effectiveness of CMTs and NTS in pathogen detection. Among the analyzed samples, 260 cases (91.6%) tested positive by NTS, and 208 cases (73.2%) tested positive by CMTs (Table 2). Notably, 8 cases (2.8%) were negative in both methods, and 192 cases (67.6%) were concordantly positive in both methods. However, 24 cases (8.5%) were exclusively detected as negative by NTS, whereas 76 cases (26.8%) were exclusively detected as negative by CMTs. The agreement analysis revealed 39 cases (13.7%) with full concordance, 143 cases (50.4%) with partial concordance, and 10 cases (3.5%) with discrepancies, highlighting significant differences between the two detection methods (Figure 2(a)).

**Table 2:** Comparison of positive rate between NTS assay and CMTs (*n* [%]).

Detection method	Positive (%)	Negative (%)	Total (%)
NTS	260 (91.55)	24 (8.45)	284 (100)
CMTs	208 (73.24)	76 (26.76)	284 (100)

Figure 2Comparison of NTS and CMTs diagnostic performance. (a) The distribution of test results across NTS and CMTs, including positive (NTS+ and CMTs+), negative (NTS− and CMTs−), and overlapping categories (Double+ and Double−). The inset highlights the classification of matched, partly matched, and mismatched cases. (b) Diagnostic performance of NTS vs. CMTs (clinical diagnosis as reference; *N* = 284). Bars show point estimates with 95% CIs. Blue = NTS; red = CMTs.

**Figure:**
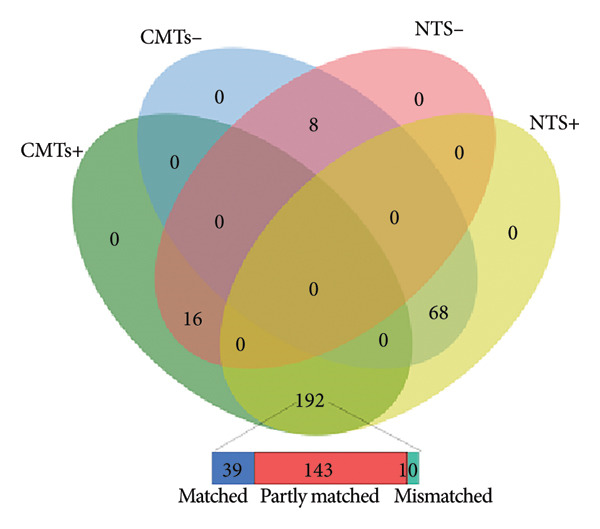
(a)

**Figure:**
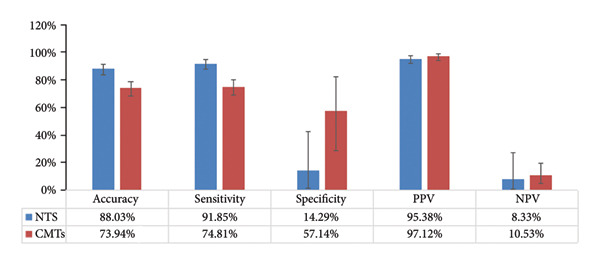
(b)

The diagnostic performance is summarized in Figure 2(b). For NTS, sensitivity was 91.85%, specificity was 14.29%, accuracy was 88.03%, PPV was 95.38%, and NPV was 8.33%. For CMTs, sensitivity was 74.81%, specificity was 57.14%, accuracy was 73.94%, PPV was 97.12%, and NPV was 10.53%. Thus, NTS yielded higher sensitivity and overall accuracy, whereas CMTs demonstrated higher specificity; PPV was high and NPV low for both tests, consistent with the high disease prevalence. Paired McNemar comparison result showed a significant difference in overall accuracy favoring NTS (*p* = 2.09 × 10^5^) and a significant sensitivity advantage for NTS (*p* = 1.68 × 10^7^), while specificity did not differ significantly between methods (*p* = 0.114) (Table S1).

### 6.1. NTS Unveils Diverse Pathogens in Pulmonary Infections

In this study, we analyzed the pathogens in 284 patients with pulmonary infections, identifying a wide range of bacteria, fungi, viruses, and other microorganisms using NTS (Figure 3). The most frequently detected bacterial pathogens included *Enterococcus faecalis* (219 cases), *Rothia mucilaginosa* (218 cases), and *Streptococcus mitis* (184 cases) (Figure 3(a)). Among fungal pathogens, *Candida albicans* (154 cases) and *Pneumocystis jirovecii* (45 cases) were predominant (Figure 3(b)). Viral pathogens such as human betaherpesvirus 7 (63 cases) and human gammaherpesvirus 4 (58 cases) and SARS‐CoV‐2 (32 cases) were also identified (Figures 3(c) and 3(d)). The diversity of pathogens highlights the complexity of pulmonary infections and the importance of comprehensive diagnostic approaches.

Figure 3Distribution of detected microorganisms across different pathogen categories. (a) Most prevalent bacterial species identified in this cohort (Top50). (b) Most prevalent fungal species identified in this cohort (Top10). (c) DNA viruses identified in this cohort (Top10). (d) RNA viruses identified in this cohort (Top10).

**Figure:**
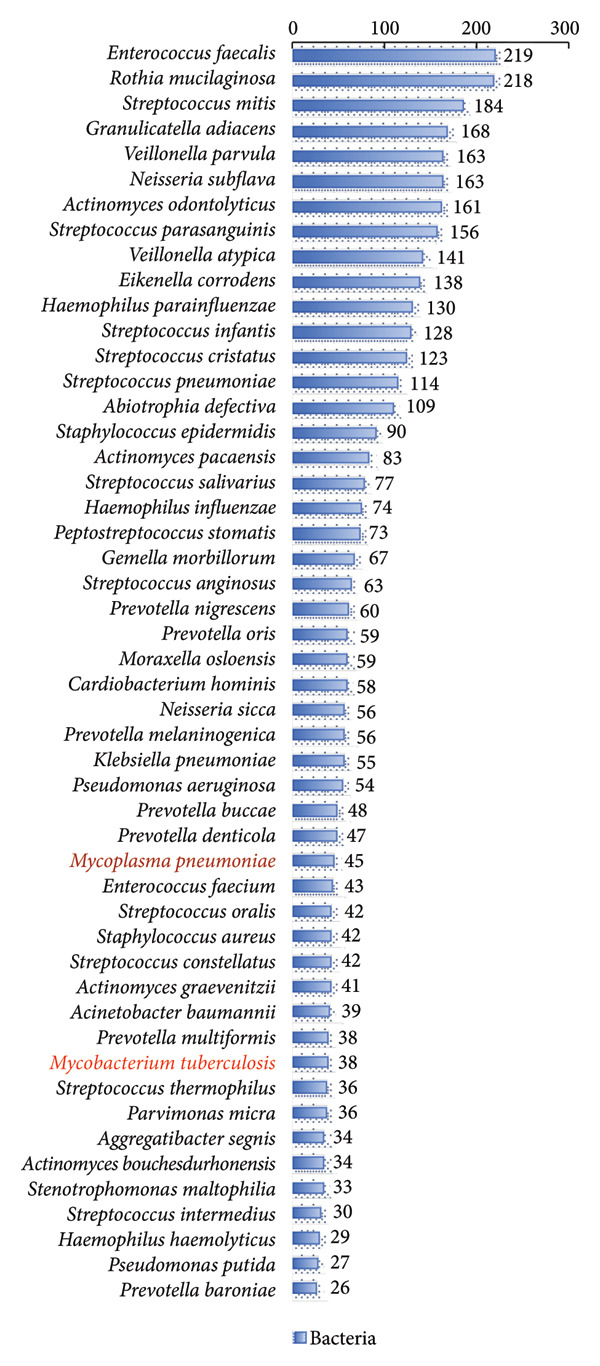
(a)

**Figure:**
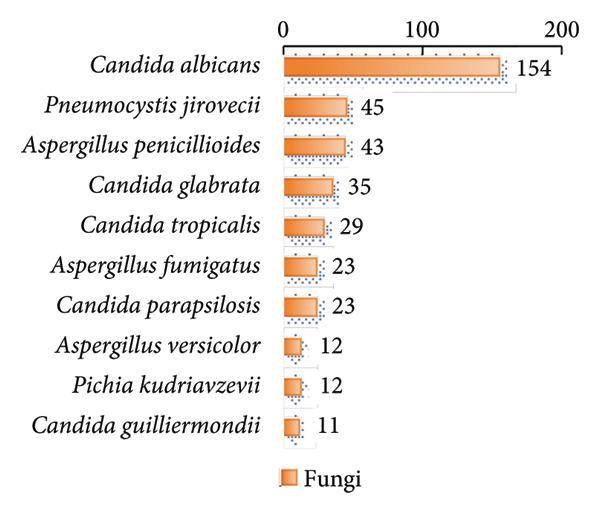
(b)

**Figure:**
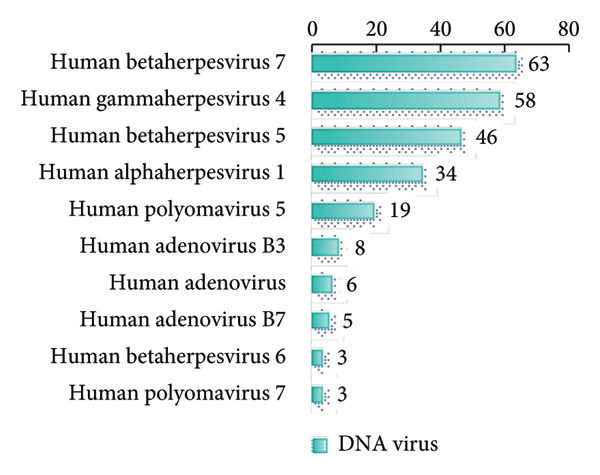
(c)

**Figure:**
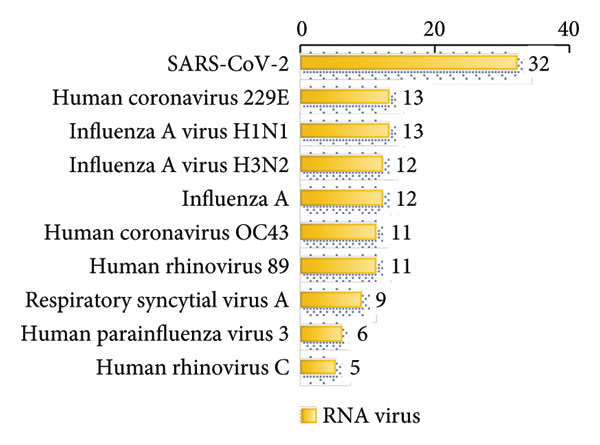
(d)

The NTS analysis revealed a broad spectrum of bacterial pathogens. Gram‐positive bacteria were the most common, with *Enterococcus faecalis*, *Rothia mucilaginosa*, and *Streptococcus mitis* being the top three most frequently identified. Other notable Gram‐positive bacteria included *Granulicatella adiacens* and *Veillonella parvula*. GNB were also identified, with *Acinetobacter odontolyticus* and *Haemophilus influenzae* among the more common ones. The presence of both Gram‐positive bacteria and GNB underscores the need for broad‐spectrum initial empiric therapy in pulmonary infections (Figure 3(a)). Fungal pathogens were also prevalent in the patient cohort. *Candida albicans* was the most frequently detected fungal pathogen, followed by *Pneumocystis jirovecii* and *Aspergillus penicillioides*. Other fungi such as *Candida glabrata* and *Candida tropicalis* were also identified. The detection of these fungi highlights the importance of considering fungal infections in patients with pulmonary symptoms, particularly in immunocompromised individuals (Figure 3(b)). Viral pathogens were identified in a significant number of patients. Human betaherpesvirus 7 and human gammaherpesvirus 4 were the most common, followed by human betaherpesvirus 5 and human alphaherpesvirus 1. Other viruses such as SARS‐CoV‐2 and human polyomavirus 5 were also detected (Figures 3(c) and 3(d)). The identification of these viruses suggests that viral infections play a substantial role in pulmonary infections and should be considered in the differential diagnosis. In addition to bacteria, fungi, and viruses, other microorganisms such as *Mycoplasma pneumoniae* (45 cases) were identified. These findings emphasize the diverse etiology of pulmonary infections and the value of NTS in detecting a wide range of pathogens. The identification of a wide variety of bacterial, fungal, viral, and other microbial pathogens underscores the complexity of these infections and the importance of accurate and rapid diagnostic methods to guide appropriate treatment.

### 6.2. Comparison of Fungal Detection Among NTS, CMTs, and Clinical Diagnosis

**Figure 4:**
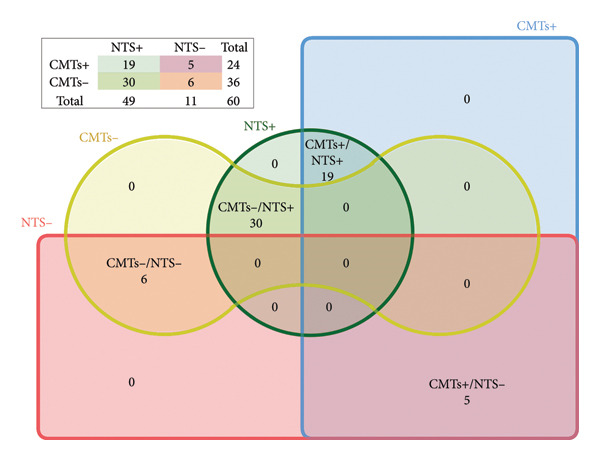
Venn diagram of fungal detection results across NTS, CMTs, and clinical diagnosis.

A total of 284 patients were included, among whom 60 were clinically diagnosed with fungal infections. NTS alone detected 153 positives, of which 49 were true positives. The sensitivity was 81.7% (49/60), specificity was 53.6% (120/224), PPV was 32.0% (49/153), and NPV was 91.6% (120/131). CMTs alone detected 42 positives, of which 24 were true positives. The sensitivity was 40.0% (24/60), specificity was 92.0% (206/224), PPV was 57.1% (24/42), and NPV was 85.1% (206/242) (Figure 4 and Table 3). Combined NTS + CMTs (defined as positive if either method was positive) detected 163 positives, of which 54 were true positives. The sensitivity was 90.0% (54/60), specificity was 51.3% (115/224), PPV was 33.1% (54/163), and NPV was 95.0% (115/121). NTS achieved higher sensitivity than CMTs but at the cost of reduced specificity and PPV. CMTs alone were highly specific but lacked sensitivity. Combined testing markedly improved sensitivity (90.0%) and NPV (95.0%), reducing the chance of missed diagnoses, but this came at the expense of lower specificity (51.3%) and PPV (33.1%), due to the accumulation of false positives when either test was considered sufficient for positivity. Therefore, the diagnosis of fungal infection cannot rely on a single test result; it must be made cautiously on the basis of comprehensive evaluation, avoiding “test‐driven” misdiagnosis and overtreatment.

**Table 3:** Diagnostic performance comparison in fungal infection of NTS, CMTs, and combined testing compared with clinical diagnosis.

Method	Sensitivity (%)	Specificity (%)	PPV (%)	NPV (%)
NTS	81.7	53.6	32.0	91.6
CMTs	40.0	92.0	57.1	85.1
NTS + CMTs	90.0	51.3	33.1	95.0

### 6.3. Diagnostic Efficacy of NTS in Mixed Infections

We present the comparison of detection rates for mixed infections identified by NTS and traditional microbial culture methods. The analysis focused on two categories: infections involving two or more species and those involving two or more types (Table 4). NTS detected mixed infections with two or more species in 283 cases (99.65%), significantly higher than traditional culture methods, which identified such infections in only 16 cases (5.63%). The *χ* ^2^ test yielded a value of 499.68, with a *p* value less than 0.001, indicating a statistically significant difference between the two methods. For mixed infections involving two or more types, NTS identified these in 278 cases (97.89%), compared to traditional culture methods that detected them in 12 cases (4.23%). These results demonstrate that NTS has a markedly higher detection rate for mixed infections compared to traditional microbial culture methods. The significant differences observed (*p* < 0.001) underscore the enhanced sensitivity and comprehensiveness of NTS in identifying complex infection profiles, which is crucial for accurate diagnosis and appropriate therapeutic interventions.

**Table 4:** Comparison of the detection rate of mixed infection between NTS and traditional microbial culture (*n* [%]).

Pathogen	NTS	Traditional culture	*X* ^2^	*p*
2 or more species	283 (99.65%)	16 (5.63%)	499.68	*p* < 0.001
2 or more types	278 (97.89%)	12 (4.23%)	494.76	*p* < 0.001

### 6.4. Microorganism Distribution in CAP and COPD

**Figure 5:**
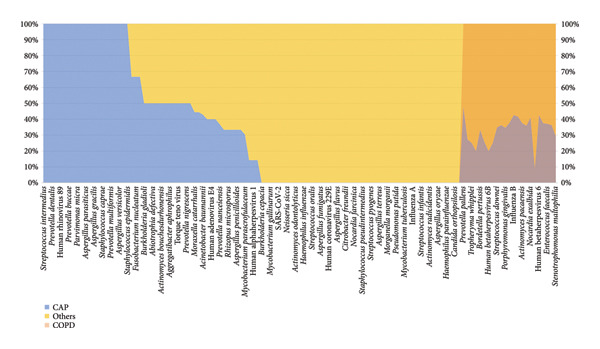
The comparative analysis of pathogen distribution across three categories of pulmonary infections: CAP (blue region); COPD (orange region).

In this study, we confirmed that the pathogen spectrum varied among different clinical types of pulmonary infection (Figure 5). In CAP, the main pathogens were bacteria and viruses. The frequently detected bacterial species were *Streptococcus pneumoniae, Haemophilus influenzae, Prevotella melaninogenica,* and *Veillonella parvula*. Viral pathogens included influenza A virus, human rhinovirus 89, and human metapneumovirus.

The relatively high proportion of viruses indicates that bacterial‐viral coinfection is a major feature of CAP. In contrast, infections in COPD were mainly bacterial and fungal. The dominant bacterial species were *Pseudomonas aeruginosa, Klebsiella pneumoniae,* and *Staphylococcus aureus*, while *Aspergillus fumigatus, Aspergillus flavus,* and *Candida albicans* were the most common fungal pathogens.

A few slow‐growing and opportunistic organisms such as *Mycobacterium tuberculosis* and *Nocardia farcinica* were also found, consistent with the chronic and recurrent nature of infection in COPD. Several species, such as *Haemophilus influenzae, Streptococcus mitis,* and *Veillonella atypica*, appeared in both groups, suggesting a shared core airway microbiota. Overall, CAP was characterized mainly by bacterial‐viral coinfections, whereas COPD was dominated by bacterial‐fungal coinfections.

### 6.5. Microorganism Distribution in Patients With and Without HBP

**Figure 6:**
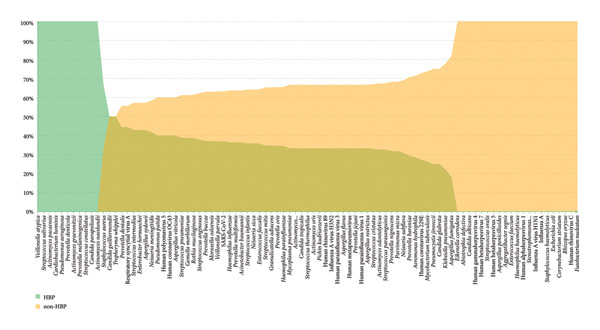
The comparative analysis of pathogen distribution across HBP patients (light green region) and non‐HBP patients (yellow region). The middle merge region contains pathogens detected in both HBP and non‐HBP.

To characterize the distinct microbial profiles of pulmonary infections between patients with and without HBP, we compared pathogen distributions in the HBP and non‐HBP groups. The results revealed differences in pathogen prevalence between the two groups (Figure 6).

In the HBP group, commensal anaerobic bacteria were markedly enriched, including *Veillonella atypica, Streptococcus parasanguinis,* and *Granulicatella adiacens.* In contrast, non‐HBP patients showed higher detection rates of opportunistic and viral pathogens, such as *Pseudomonas aeruginosa, Acinetobacter baumannii, Klebsiella pneumoniae, Aspergillus fumigatus,* influenza A virus, and human coronavirus 229E. Some species, including *Haemophilus influenzae* and *Streptococcus pneumoniae*, were common to both groups.

These results indicate both shared and distinct microbial characteristics between hypertensive and nonhypertensive individuals. Hypertensive patients tended to harbor increased proportions of oral‐derived commensals, while nonhypertensive patients were more susceptible to opportunistic bacterial and viral infections. This difference suggests that HBP may influence airway microbial composition and highlights the importance of considering underlying conditions such as HBP in the clinical management of pulmonary infections.

## 7. Discussion

This study systematically analyzed the distribution of pathogens and the diagnostic performance of NTS compared with CMTs in 284 patients with pulmonary infections. Bacteria were the predominant pathogens, followed by fungi and viruses, with CAP being the most common type. Compared with CMTs, NTS demonstrated markedly higher sensitivity and detected a wider range of pathogens, especially in mixed infections. These findings are consistent with previous studies confirming the superior diagnostic yield of NTS in respiratory and systemic infections [15, 16].

NTS effectively identified diverse bacteria, fungi, and viruses, including *Enterococcus*, *Candida*, and *Herpesviridae*, that were frequently missed by traditional culture‐based methods. Its broad‐spectrum detection capability enables earlier diagnosis and more accurate antimicrobial therapy. Although CMTs retain high specificity, their dependence on culture limits detection of slow‐growing or low‐abundance pathogens. The combined use of both methods can enhance diagnostic accuracy, reduce false negatives, and improve etiological precision.

In this study, NTS also proved valuable for distinguishing pathogen profiles across pulmonary infection types. In CAP, the detection rate of viral pathogens such as influenza A and parainfluenza virus was significantly higher with NTS, suggesting that bacterial‐viral coinfections are common [17]. Early identification of viral infections is crucial for optimizing treatment, reducing unnecessary antibiotic use, and promoting targeted antiviral therapy. In contrast, infections associated with COPD were predominantly bacterial‐fungal. The main bacterial pathogens included *Pseudomonas aeruginosa, Klebsiella pneumoniae,* and *Staphylococcus aureus*, accompanied by opportunistic fungi such as *Aspergillus fumigatus* and *Candida orthopsilosis*[18]. These findings align with prior evidence that COPD patients are prone to polymicrobial colonization and secondary infections due to chronic airway inflammation, mucus retention, and impaired clearance [6, 7].

NTS further revealed distinct microbial patterns among patients with and without HBP (HBP vs. non‐HBP). In hypertensive individuals, commensal anaerobes such as *Veillonella atypica, Streptococcus parasanguinis,* and *Granulicatella adiacens* were more abundant, suggesting an altered airway microbial ecosystem. In contrast, nonhypertensive patients exhibited higher frequencies of opportunistic and viral pathogens, including *Pseudomonas aeruginosa, Acinetobacter baumannii, Klebsiella pneumoniae,* influenza A virus, and human coronavirus 229E. Shared respiratory pathogens such as *Haemophilus influenzae* and *Streptococcus pneumoniae* were detected in both groups, whereas *Veillonella atypic*a was dominant among hypertensive patients. These results indicate that HBP may influence airway microbial composition, possibly through immune dysregulation or systemic inflammation, as also suggested by other studies [19, 20].

Importantly, NTS not only identified classical pathogens but also provided ecological insights into the airway microbiota. The coexistence of pathogens and commensal organisms implies that pulmonary infections represent ecological disturbances rather than isolated infectious events. The microbial imbalance observed in CAP, COPD, and hypertensive patients likely reflects both pathogenic processes and host‐microbiota interactions. This concept supports the growing view that infection susceptibility is closely linked to microbial dysbiosis, rather than the presence of a single dominant pathogen [21, 22].

Despite its advantages, NTS has limitations. Its high sensitivity can lead to false positives due to the detection of nonpathogenic or contaminant organisms. Therefore, interpretation requires correlation with clinical and radiological findings. In contrast, while CMTs exhibit higher specificity, they are often limited by false negatives, particularly for anaerobes, mycobacteria, or fungi with specific growth requirements. Furthermore, CMTs are less effective for viral detection, which is essential for CAP and other pulmonary infections [23]. Consequently, combining NTS and CMTs offers complementary strengths: NTS provides broad‐spectrum screening, while CMTs confirm clinical relevance and resistance profiles.

This study has several limitations. Being a single‐center study with a moderate sample size, generalizability may be limited. Future multicenter investigations with larger cohorts are necessary to validate these findings. Moreover, patient heterogeneity, including immune status, may influence microbial distribution and should be considered in subsequent research.

## 8. Conclusion

NTS provides higher sensitivity, broader pathogen coverage, and superior detection of complex infections compared with CMTs. It is particularly useful for the diagnosis of CAP, COPD, and other polymicrobial pulmonary infections. Furthermore, by identifying microbial differences between hypertensive and nonhypertensive patients, NTS expands understanding of host‐microbiota interactions and comorbidity‐related susceptibility. Although cost and technical demands currently limit its use, advances in sequencing efficiency and affordability are improving accessibility [19, 22]. With a turnaround time of approximately 48 h, substantially faster than traditional cultures, NTS facilitates rapid diagnosis and targeted therapy. Its integration into clinical workflows is expected to enhance pathogen detection, guide precise antimicrobial strategies, and contribute to the advancement of precision medicine.

## Ethics Statement

Ethical approval was granted by the Ethics Committee of Chifeng Clinical College of Inner Mongolia Medical University (CK20250501).

## Disclosure

All authors reviewed and approved the final manuscript.

## Conflicts of Interest

The authors declare no conflicts of interest.

## Author Contributions

Ying Zhao designed the study, performed data collection, and wrote the manuscript. Guannan Ma performed data collection, conducted laboratory experiments, and wrote the manuscript. Tianxiao Ma collected clinical data. Yongshuai Miao and Heya Na participated in sample processing. Yuhui Liu conducted data analysis. Xiaohua Li designed the study, supervised the research, and edited the manuscript. Ying Zhao and Guannan Ma are co‐first authors of this article and contributed equally to this article.

## Funding

This study was supported by the Natural Science Foundation of Chifeng (SZR24075).

## Supporting Information

Detailed procedure of NTS testing. NTS testing: Respiratory tract samples were collected in sterile sputum containers and immediately transported on dry ice to a commercial laboratory (Hangzhou DIAN Medical Laboratory, Hangzhou, China) for testing.

Table S1: Detailed indicators of diagnostic performance.

## Supporting information


**Supporting Information** Additional supporting information can be found online in the Supporting Information section.

## Data Availability

The data that support the findings of this study are available on request from the corresponding author. The data are not publicly available due to privacy or ethical restrictions.
