# Supplementary material for: Assessing Nanopore Targeted Sequencing for the Diagnosis of Pulmonary Infections: A Comparative Multidisease Approach
Source: Can J Infect Dis Med Microbiol. 2026 Feb 3;2026:5479737. doi: 10.1155/cjid/5479737 (PMC12868382; doi:10.1155/cjid/5479737)
Supplement: Supplementary file 1 — Supporting Information Additional supporting information can be found online in the Supporting Information section. [file CJID-2026-5479737-s001.zip › 5479737.f1.docx]

**Supplementary 1. Detailed procedure of NTS testing**

NTS testing: Respiratory tract samples were collected in sterile sputum containers and immediately transported on dry ice to a commercial laboratory (Hangzhou Dean Medical Laboratory, Hangzhou, China) for testing.

**1. Sample Preprocessing**

Sputum and broncho-alveolar-lavage (BALF) specimens were liquefied by mixing 1:1 (v/v) with liquefaction reagent, vortexing, and leaving at room temperature for 5 min until no macroscopic clumps remained; tubes were then briefly centrifuged. Oral-swab tips were immersed in 1 × PBS. All samples were centrifuged at 12 000 × g for 5 min. Turbid biological fluids: the supernatant was discarded, leaving 300 µL; 51 µL of extraction-enhancer cocktail was added, vortexed, and incubated at 37 °C for 15 min. Clear biological fluids: the supernatant was removed, leaving 500 µL; 51 µL of extraction-enhancer cocktail was added, vortexed, and incubated at 37 °C for 10 min. Turbid fluids subsequently received 200 µL 1 × PBS plus 20 µL proteinase K, followed by shaking incubation at 56 °C, 1 000 rpm for 10 min. After vortexing and a short spin, the entire mixture was transferred to a bead-beating tube, which was tightly capped.

**2. Nucleic acid extraction**

Host DNA was depleted with a selective lysis/adsorption kit prior to microbial DNA purification as described below. DNA concentration was measured using a Qubit 3.0 fluorometer.

**3. Library preparation**

**3.1 Reverse transcription and PCR amplification、**

RNA was reverse-transcribed, followed by PCR amplification. Amplified products were purified with magnetic beads, washed with 70–80% ethanol, eluted in ddH2O, and quantified.

**3.2 End repair and barcoding**

DNA fragments were treated with end-repair enzymes, ligated with barcodes, pooled, purified with magnetic beads, and washed with 1.5 * SFB solution and ethanol. DNA was eluted in water and quantified.

**3.3 Adapter ligation**

Barcoded libraries were ligated with adapters, purified, washed, and eluted in EB buffer. Final concentrations were measured prior to sequencing.

**4. Machine sequencing**

Sequencing buffer (SQB), loading beads (LB), flush buffer (FB), and flush tether (FLT) were equilibrated at room temperature. The MinION flow cell was primed with FB/FLT mix, and the sequencing library was prepared with 37.5 μL SQB, 25.5 μL LB, and 40–80 ng DNA in a total of 75 μL. Libraries were loaded onto the GridION Mk1 for nanopore sequencing following standard procedures.

| Table S1. Detailed indicators of diagnostic performance. | | | | |
| --- | --- | --- | --- | --- |
| **Method** | **Metric** | **Estimate** | **Lower** | **Upper** |
| NTS | Sensitivity | 0.918518519 | 0.879234019 | 0.948232452 |
| NTS | Specificity | 0.142857143 | 0.017794515 | 0.428129161 |
| NTS | PPV | 0.953846154 | 0.920766967 | 0.975927416 |
| NTS | NPV | 0.083333333 | 0.010256341 | 0.269972802 |
| NTS | Accuracy | 0.88028169 | 0.836739745 | 0.915649507 |
| NTS | Kappa | 0.045849802 |  |  |
| CMTs | Sensitivity | 0.748148148 | 0.691962084 | 0.798797 |
| CMTs | Specificity | 0.571428571 | 0.2886094 | 0.82338891 |
| CMTs | PPV | 0.971153846 | 0.938274495 | 0.989341904 |
| CMTs | NPV | 0.105263158 | 0.046553563 | 0.196916699 |
| CMTs | Accuracy | 0.73943662 | 0.684289955 | 0.789510342 |
| CMTs | Kappa | 0.103106862 |  |  |
| NTS vs CMTs | McNemar_overall_p | 2.08817E-05 |  |  |
| NTS vs CMTs | McNemar_sensitivity_p | 1.68464E-07 |  |  |
| NTS vs CMTs | McNemar_specificity_p | 0.113846298 |  |  |
